# Integrating Community Health Workers Into Clinical Settings

**DOI:** 10.1111/hex.70604

**Published:** 2026-02-18

**Authors:** Pearl A. McElfish, Sara Sorrell, Luis Paganelli Marin, Judy Pile, Anna Huff, Bonnie Faitak, Sarah Moore, Amy Ayala, Sergio Bonilla, Carolina N. Vargas, Manuel E. Tejada, Nicole Thornton, Krista Langston

**Affiliations:** ^1^ Institute for Community Health Innovation University of Arkansas for Medical Sciences Northwest Springdale Arkansas USA; ^2^ College of Medicine University of Arkansas for Medical Sciences Northwest Springdale Arkansas USA; ^3^ Arkansas Community Health Workers Association Little Rock Arkansas USA; ^4^ Arkansas Center for Women and Infants' Health University of Arkansas for Medical Sciences Northwest Little Rock Arkansas USA

**Keywords:** clinical settings, Community Health Workers, integration

## Abstract

**Introduction:**

As Medicaid policies continue to evolve to support Community Health Worker (CHW) services, it is important to understand the facilitators and barriers to integrating CHWs into a clinical team. This study used parallel multi‐methods to understand facilitators and barriers to CHW integration.

**Methods:**

Using parallel, multi‐methods, we conducted surveys and focus groups with CHWs recruited from Arkansas. CHWs were asked to complete a survey and invited to take part in a focus group. Additional focus group participants were recruited in person and asked to complete a survey after the focus group. The survey captured respondents' demographic characteristics, employer characteristics, and items from the CHW Common Indicators Project. The focus group guide included core questions and probes to understand CHWs' perceptions of barriers and facilitators to integration into the clinical setting.

**Results:**

A total of 57 CHWs participated in the focus groups, with 53 completing the survey. In the quantitative surveys, CHWs reported a high level of support and integration. Focus groups provided an opportunity for CHWs to discuss factors that increased or hindered their successful integration. These themes included: (1) Role Clarity, (2) Understanding Community Needs, (3) Expectations versus Reality, (4) Experiences of Onboarding into a New Organization, (5) Open Communication with Supervisors, and (6) Comradery, Collaboration, and Co‐Learning.

**Conclusions:**

By identifying the factors that support or hinder effective CHW integration, these findings can inform organizational policies and strategies aimed at optimizing CHW contributions within clinical settings.

**Patient or Public Contribution:**

More than half of the authors are Community Health Workers (CHWs) and CHW supervisors. These CHWs and CHW supervisors were involved in the study design, including data collection, data analysis, data interpretation, and manuscript authorship. This approach ensures the voices of CHWs and their supervisors are integrated into every aspect of the article and allows for detailed insight from CHWs in their own words, which is essential for understanding the complex real‐world dynamics influencing the integration of CHWs. This approach also allows for practical recommendations for improving the integration of CHWs.

## 1 Introduction

Residents of Arkansas experience significant health disparities compared to the national average, including disparate rates of obesity, heart disease, diabetes, and cancer, and some of the highest rates of maternal and infant mortality [1, 2, 3, 4, 5, 6, 7]. Racially and ethnically minoritized residents, residents of rural communities, and residents of low‐income communities face even starker disparities in both healthcare access and health outcomes [8, 9, 10, 11, 12]. These disparities stem from a combination of socioeconomic, cultural, and systemic factors that disproportionately impact rural, racially/ethnically minoritized, and low‐income populations [13, 14, 15, 16, 17, 18, 19]. Arkansas is a racially/ethnically diverse state, and approximately 16% of the residents live in poverty (compared to 12.4% in the United States [US]) [20]; 44% of the population resides in rural areas [21]; most of the counties are medically underserved; and 15 of the counties are partially underserved [22, 23]. The limited resources and limited access often result in delayed or insufficient healthcare services [23].

Community Health Workers (CHWs) serve a crucial role in bridging healthcare systems and the communities they serve, particularly in underserved areas [24, 25, 26, 27, 28, 29, 30, 31]. CHWs often work with and report to registered nurses and/or social workers and have varied job duties, including screening for social determinants of health, making referrals, helping patients navigate the health system, assisting with care plans, and providing health education [32].

CHWs often share cultural, linguistic, and social ties with the communities they serve, enabling them to effectively deliver culturally competent and contextually appropriate care [27, 28, 29, 33]. Several systematic reviews have demonstrated that CHWs can help increase preventive care utilization, improve chronic disease management, and reduce healthcare costs [24, 25, 26, 27, 28, 34, 35, 36]. Additionally, CHWs have been shown to enhance patient engagement and adherence to treatment plans by fostering trust between healthcare providers and marginalized populations [26].

Given the value of CHWs, their integration into primary care settings has increased in recent years [37], a trend supported by evolving Medicaid policies [38, 39]. Many states have expanded Medicaid reimbursement to include CHW‐provided services [40, 41]. As of April 2025, 29 states allow Medicaid reimbursement for CHW services, through state plan amendments, 1115 demonstration waivers, managed care organizations, or a combination of these approaches [42, 43]. Arkansas recently passed HB1258 to establish a statewide certification for CHWs and provide guidelines for reimbursement [44].

Despite evidence of their effectiveness and increasing reimbursement policies, integrating CHWs into health care teams remains complex and understudied. The limited literature focusing on CHW integration has identified multiple barriers influencing the successful integration of CHWs into clinical teams [45]. Prior research has documented ambiguity and a lack of understanding surrounding CHWs' roles and responsibilities, as well as challenges interacting with the broader health care team [46, 47]. Among the most commonly cited barriers was the lack of understanding of CHW roles [37, 38, 45, 46, 47, 48, 49].

In 2016, Tri County, a local nonprofit organization in Arkansas, began implementing CHW training that incorporated the C3 competencies [50]. In 2020, the University of Arkansas for Medical Sciences and Rural Health Partnership also begin training CHWs [51]. There are approximately 400 CHWs trained in Arkansas, and the number is anticipated to increase over the next few years. Current training programs include both a standard training track and an apprenticeship program [51].

As Medicaid policies continue to evolve to support CHW services, it is important to understand factors affecting the integration of CHWs into a clinical team. This study used parallel multi‐methods to understand facilitators and barriers to CHW integration. By identifying the factors that support or hinder effective CHW integration, these findings can inform organizational policies and strategies aimed at optimizing CHW contributions within clinical settings.

## 2 Methods

### 2.1 Study Design

Using parallel multi‐methods, which involves collecting and analyzing qualitative and quantitative data simultaneously in order to compare and contrast the findings of each, we conducted a survey and focus groups with CHWs [52, 53]. CHWs were recruited from the Arkansas Community Health Worker Association and the University of Arkansas for Medical Sciences Institute for Community Health Innovation e‐mail listservs. Because this listserv is used for training and monthly meetings, there is robust participation from CHWs across the state. CHWs who are part of the listserv work for a variety of organizations including nonprofits, primary care clinics including federally qualified health centers, and the state's university medical center with primary care locations around the state. CHWs were asked to complete a survey in REDCap [54] and were invited to take part in a focus group. Additional focus group participants were recruited in person at the annual Arkansas Community Health Worker Association Summit and asked to complete the survey after their focus group. The survey captured respondents' demographic characteristics, employer characteristics, and items from the CHW Common Indicators Project [55]. Common indicators for CHWs focus on capturing both how services are delivered and whether those services lead to meaningful change, reflecting the CHW role as a bridge between communities and health systems. These measures emphasize engagement, trust, and successful linkage to care and resources because these mechanisms are central to how CHWs influence behaviors, access, and downstream health outcomes. Respondents were offered a $25 gift card for their participation in the survey and a $40 gift card for their participation in the focus group. Participants gave electronic consent for the survey and verbal consent for the qualitative interviews. The study was approved as exempt by the University of Arkansas for Medical Sciences Institutional Review Board (#275076) and took place between 1 March, 2024, and 30 June, 2024.

### 2.2 Quantitative Survey

Demographic characteristics captured in the survey included age, gender, race/ethnicity, sexual orientation, highest level of educational attainment, number of languages spoken, length of time working as a CHW, and whether CHWs lived within the communities they serve. Employer characteristics included organization type, compensation offered, and benefits offered. Several survey items from the CHW Common Indicators Project [55] were included on the survey with Likert‐type responses. Descriptive statistics were generated using Stata version 18.5 [56].

### 2.3 Qualitative Interviews

Interviews were facilitated in person by two qualitative researchers and used a semi‐structured focus group guide to encourage consistency in discussions across focus groups. The focus group guide included core questions and probes to understand CHWs' perceptions of the barriers and facilitators to integration into the clinical setting. Sample questions included, ‘How well do you think your supervisor understands your role in your team and/or organization?’ and ‘Are there policies or processes which make it difficult to do your job?’. Focus groups lasted between 60 and 90 min. The focus groups were audio‐recorded and transcribed.

### 2.4 Qualitative Data Analysis

Two researchers analyzed the qualitative data using thematic analysis following established methodological guidance [57, 58]. First, the researchers familiarized themselves with the data through repeated reading of transcripts. Then, researchers systematically coded the data and collated related codes into possible themes. The researchers interpreted and synthesized the themes to develop the codebook of emergent themes. All data were coded using the codebook and identifying exemplar quotes and inclusion and exclusion criteria for each code. Data was then coded by two coders with confirmation from two additional coders. Coded texts were reviewed, refined, and clearly defined to ensure coherence and relevance. Discrepancies in coding were discussed and resolved using consensus. Direct quotes are shared verbatim to most accurately represent CHW participants in their own words.

## 3 Results

### 3.1 Quantitative Results

A total of 57 CHWs participated in the focus groups, with 53 completing the REDCap survey. Four participants left after the focus group and before completing the survey due to time constraints. Half of the participants (49.1%) were between the ages of 35 and 54, and the majority (84.9%) were female. Participants represented diverse racial/ethnic groups, including Native Hawaiian or Pacific Islander (34.0%), Black or African American (22.6%), Non‐Hispanic White (22.6%), and Hispanic or Latino (17.0%). The majority of participants reported completing some education beyond high school (83.0%). Approximately two‐thirds (69.8%) spoke multiple languages and reported working as a CHW between 1 and 5 years (62.3%). The vast majority (90.6%) reported living within the community they serve (Table 1).

**Table 1:** Demographic characteristics of CHWs responding to CHW integration survey (*n* = 53).

	*N* (%)
Age category	
18–34	19 (35.8%)
35–54	26 (49.1%)
55+	8 (15.1%)
Gender	
Male	8 (15.1%)
Female	45 (84.9%)
Other	0 (0%)
Race/Ethnicity	
Hispanic or Latino	9 (17.0%)
White	12 (22.6%)
Black or African American	12 (22.6%)
American Indian or Alaska Native	2 (3.8%)
Native Hawaiian or Pacific Islander	18 (34.0%)
Asian	0 (0%)
Sexual orientation	
Heterosexual or straight	47 (88.7%)
Bisexual	4 (7.5%)
Lesbian or gay	0 (0%)
Other	0 (0%)
Declined to answer	2 (3.8%)
Highest education	
High school or less	9 (17.0%)
Some college or associate degree	28 (52.8%)
Bachelor's degree	12 (22.6%)
Postgraduate degree	4 (7.5%)
Speak multiple languages	
No	16 (30.2%)
Yes	37 (69.8%)
Length of work as CHW	
< 1 y	8 (15.1%)
1 y−5 y	33 (62.3%)
5+ y	12 (22.6%)
Live within community served	
No	5 (9.4%)
Yes	48 (90.6%)

More than half of the sample (58.5%) reported their employer was an academic medical center/research institution, with nearly a quarter (22.6%) reporting their employer was a community‐based organization/community‐based clinic and 13.2% reporting working for a federally qualified health center (Table 2).

**Table 2:** Workplace characteristics reported by CHWs responding to CHW integration survey (*n* = 53).

	*N* (%)
Organization type	
Federally Qualified Health Center	7 (13.2%)
Academic Medical Center/Research Institute	31 (58.5%)
Community‐Based Organization/Community‐Based Clinic	12 (22.6%)
Other	3 (5.7%)
Compensation type	
Salary	38 (73.1%)
Hourly	14 (26.9%)
Benefits offered	
Health insurance	46 (88.5%)
Dental insurance	43 (82.7%)
Disability insurance	26 (50.0%)
Mental health insurance	11 (21.2%)
Paid family leave	22 (42.3%)
Paid sick leave	40 (76.9%)
Paid vacation	42 (80.8%)
Transport or mileage reimbursement	43 (82.7%)
Cell phone subsidy/reimbursement	18 (34.6%)
Internet subsidy/reimbursement	3 (5.8%)
Employee assistance program	18 (34.6%)
Retirement program	27 (51.9%)
Bonuses	10 (19.2%)
Hazard pay	1 (1.9%)
Overtime pay	8 (15.4%)
Education reimbursement/stipend	16 (30.8%)
Cost of living Adjustment	0 (0.0%)
Professional development funds	13 (25.0%)
Professional development opportunities	22 (42.3%)
No benefits offered	1 (1.9%)

CHW responses to survey items adapted from the Common Indicators Project are shown in Table 3. The majority of respondents agreed or strongly agreed they understood their role and scope of work within their team (98.1%), their team saw the value in what they do (96.3%), they had received the right amount (88.7%) and type (92.4%) of training to be an effective CHW, their team received training on the importance of CHWs (94.3%), and their team understood their role and scope of work (92.4%). Regarding their employers, the majority agreed or strongly agreed they were provided the tools needed to be an effective CHW (90.6%), their employer's protocols and workflows were documented (92.5%), and there were standard operating procedures for when and how they work with patients/clients (92.4%). The majority also reported membership in groups that influence policy in their employing organization (64.1%) and having influenced policy within their organization (69.8%). Concerning supervisors, the majority agreed or strongly agreed their supervisor appreciated their role as a CHW (94.3%), advocated for the role of CHWs with upper management (86.7%), participated in training about the CHW profession (84.9%), encouraged their professional growth (92.5%), and understood the strengths and needs of the communities they serve (94.3%). Additionally, the majority (54.8%) reported that CHWs participate on hiring panels when CHW supervisors are selected. Regarding other healthcare, social service, and education providers in their organization, the majority reported often or constantly communicating about clients/patients (73.6%), often or always receiving communication in a timely way about clients/patients (52.8%), others knowing some or a lot about the work they did with clients/patients (66.0%), feeling others respected them and the work they did with clients/patients a lot or completely (62.2%), feeling others understood their role and the work they do as a CHW some or a lot (62.2%), and feeling a lot or completely comfortable going to others to talk about client/patient needs (66.0%). Additionally, the majority reported feeling not at all or a little isolated due to their race/ethnicity or culture (66.1%), feeling not at all or a little that they had to be the only voice for their race/ethnicity or culture (60.4%), and feeling not at all or a little dismissed or devalued due to their racial/ethnic or cultural background (71.7%). Overall, participants indicated they felt a lot or completely integrated into their organization (62.3%), team (73.6%), and organization's workflows and processes (62.3%).

**Table 3:** Integration factors reported by CHWs responding to CHW integration survey (*n* = 53).

	*N* (%)
I understand my role and scope of work within my team.	
Strongly disagree	1 (1.9%)
Disagree	0 (0%)
Agree	13 (24.5%)
Strongly agree	39 (73.6%)
My team sees the value in what I do.	
Strongly disagree	1 (1.9%)
Disagree	1 (1.9%)
Agree	18 (34.0%)
Strongly agree	33 (62.3%)
I have received the right amount of training to be an effective CHW.	
Strongly disagree	1 (1.9%)
Disagree	5 (9.4%)
Agree	24 (45.3%)
Strongly agree	23 (43.4%)
I have received the right type of training to be an effective CHW.	
Strongly disagree	0 (0%)
Disagree	4 (7.5%)
Agree	27 (50.9%)
Strongly agree	22 (41.5%)
My team has received training on the importance of CHWs.	
Strongly disagree	1 (1.9%)
Disagree	2 (3.8%)
Agree	21 (39.6%)
Strongly agree	29 (54.7%)
My team understands my role and scope of work.	
Strongly disagree	1 (1.9%)
Disagree	3 (5.7%)
Agree	22 (41.5%)
Strongly agree	27 (50.9%)
My employer provides me with all of the tools I need to be an effective CHW.	
Strongly disagree	0 (0%)
Disagree	5 (9.4%)
Agree	25 (47.2%)
Strongly agree	23 (43.4%)
My employer's protocols and workflows are documented.	
Strongly disagree	1 (1.9%)
Disagree	3 (5.7%)
Agree	25 (47.2%)
Strongly agree	24 (45.3%)
There are standard operating procedures for when and how I work with patients/clients.	
Strongly disagree	2 (3.8%)
Disagree	2 (3.8%)
Agree	21 (39.6%)
Strongly agree	28 (52.8%)
I am a member of one or more groups that influence policy in my employing organization.	
Strongly disagree	4 (7.5%)
Disagree	15 (28.3%)
Agree	21 (39.6%)
Strongly agree	13 (24.5%)
I believe that as a CHW, I have influenced policy in my organization.	
Strongly disagree	3 (5.7%)
Disagree	13 (24.5%)
Agree	21 (39.6%)
Strongly agree	16 (30.2%)
My supervisor appreciates my role as a CHW.	
Strongly disagree	1 (1.9%)
Disagree	2 (3.8%)
Agree	19 (35.8%)
Strongly agree	31 (58.5%)
My supervisor advocates for the role of CHWs with upper management (staff who rank above the supervisor).	
Strongly disagree	1 (1.9%)
Disagree	6 (11.3%)
Agree	19 (35.8%)
Strongly agree	27 (50.9%)
My supervisor has participated in training about the CHW profession.	
Strongly disagree	1 (1.9%)
Disagree	7 (13.2%)
Agree	23 (43.4%)
Strongly agree	22 (41.5%)
My supervisor encourages my professional growth (e.g., by regularly encouraging me and/or accepting my suggestions within supervision sessions to pursue training opportunities, attend conferences, develop leadership skills, etc.).	
Strongly disagree	0 (0%)
Disagree	4 (7.5%)
Agree	18 (34.0%)
Strongly agree	31 (58.5%)
My supervisor understands the strengths and needs of the community/ies we serve.	
Strongly disagree	0 (0%)
Disagree	3 (5.7%)
Agree	19 (35.8%)
Strongly agree	31 (58.5%)
In my organization, CHWs participate on hiring panels when CHW supervisors are selected.	
Strongly disagree	12 (22.6%)
Disagree	12 (22.6%)
Agree	18 (34.0%)
Strongly agree	11 (20.8%)
How frequently do you communicate with the other healthcare, social service, and/or education providers in your organization about clients/patients?	
Rarely	4 (7.5%)
Occasionally	10 (18.9%)
Often	23 (43.4%)
Constantly	16 (30.2%)
Do the other healthcare, social service, and/or education providers in your organization communicate with you in a timely way about clients/patients?	
Rarely	6 (11.3%)
Occasionally	19 (35.8%)
Often	16 (30.2%)
Always	12 (22.6%)
How much do the other healthcare, social service, and/or education providers in your organization know about the work you do with clients/patients?	
Nothing	1 (1.9%)
A little	10 (18.9%)
Some	21 (39.6%)
A lot	14 (26.4%)
Everything	7 (13.2%)
How much do the other healthcare, social service, and/or education providers in your organization respect you and the work you do with clients/patients?	
Not at all	0 (0%)
A little	2 (3.8%)
Some	18 (34.0%)
A lot	20 (37.7%)
Completely	13 (24.5%)
To what extent do the other healthcare, social service, and/or education providers in your organization understand your roles and what you do as a CHW?	
Not at all	3 (5.7%)
A little	8 (15.1%)
Some	20 (37.7%)
A lot	13 (24.5%)
Completely	9 (17.0%)
To what extent do you feel comfortable going to the other healthcare, social service, and/or education providers in your organization to talk about client/patient needs?	
Not at all	1 (1.9%)
A little	4 (7.5%)
Some	13 (24.5%)
A lot	15 (28.3%)
Completely	20 (37.7%)
Do you feel isolated from the other healthcare, social service, and/or education providers in your organization because of your race/ethnicity or culture?	
Not at all	26 (49.1%)
A little	9 (17.0%)
Some	13 (24.5%)
A lot	4 (7.5%)
Completely	1 (1.9%)
Do you feel like you have to be the only voice for your race/ethnicity or culture amongst the other healthcare, social service, and/or education providers in your organization?	
Not at all	26 (49.1%)
A little	6 (11.3%)
Some	12 (22.6%)
A lot	7 (13.2%)
Completely	2 (3.8%)
Do you feel dismissed or devalued by the other healthcare, social service, and/or education providers in your organization because of your racial/ethnic or cultural background?	
Not at all	30 (56.6%)
A little	8 (15.1%)
Some	11 (20.8%)
A lot	3 (5.7%)
Completely	1 (1.9%)
To what extent do you feel integrated into your organization?	
Not at all	1 (1.9%)
A little	2 (3.8%)
Some	17 (32.1%)
A lot	24 (45.3%)
Completely	9 (17.0%)
To what extent do you feel integrated into your team?	
Not at all	0 (0%)
A little	2 (3.8%)
Some	12 (22.6%)
A lot	18 (34.0%)
Completely	21 (39.6%)
To what extent do you feel integrated into your organization's workflows and processes?	
Not at all	1 (1.9%)
A little	2 (3.8%)
Some	17 (32.1%)
A lot	24 (45.3%)
Completely	9 (17.0%)

### 3.2 Qualitative Results

The qualitative responses focused on factors influencing CHWs' integration into their organization. CHWs identified multiple factors to successfully integrating within the organizations they serve. These themes included: (1) Role Clarity, (2) Understanding Community Needs, (3) Expectations versus Reality, (4) Experiences of Onboarding into a New Organization, (5) Open Communication with Supervisors, and (6) Comradery, Collaboration, and Co‐Learning. Qualitative themes are shown in Table 4.

**Table 4:** Qualitative themes.

Qualitative findings	Recommendations for organizational policy and practice
Role clarity	Organizations should work to ensure clear job descriptions.
Understanding community needs	Organizations should find ways engage with the community.
Expectations versus reality	Organizations should develop CHW‐specific metrics for success and understand the need for flexibility.
Experiences of onboarding into a new organization	Organizations should provide an onboarding process that allows CHWs to understand their role within the organization.
Open communication with supervisors	Organizations should encourage open communication and feedback between CHWs and their organizations, while being cognizant some CHWs' cultural backgrounds may make it more difficult to provide feedback to those in authority.
Comradery, collaboration, and co‐learning	Organizations should seek ways to foster comradery, collaboration, and co‐learning.

### 3.3 Role Clarity

CHWs described barriers with a lack of clarity in their role and responsibilities. One CHW stated, ‘It's evolving. I appreciate the fact that I have a lot of leeway’. This CHW went on to say, ‘I'll be honest with you, from the day I got there, I don't think they knew what to do with me… No one has honestly told me what to do. It's evolving’. Another CHW agreed, stating, ‘I think there's a big assumption… I don't know if they're still trying to figure that out when they're giving you your role. I don't feel like there's any clarity’. This CHW went on to explain, ‘I understand that they may be, still, trying to figure it out. It's really hard to do your job and meet the goals, and you want to meet goals and objectives… Just being able to have that clarity’. Some CHWs stated they and their supervisor were learning alongside each other, as many CHW positions are part of new grant funded programs that have not been done before in the organization. A CHW explained, ‘I think that my supervisor, she does work to understand what I'm supposed to be doing. We're constantly looking at the grant and she'll ask me, “Did you see so‐and‐so, so‐and‐so in there?” I'm constantly finding things, so she's working to—we're a work in progress. That's okay’. CHWs described effective supervisors as those who understand the role of a CHW clearly. Specifically, CHWs identified having a supervisor who had been a CHW as particularly effective, stating, ‘My supervisor is also a CHW, and I think that it's so helpful to have somebody that has done the work and can see those things’.

### 3.4 Understanding Community Needs

CHWs described experiencing challenges when their supervisors and organizational leadership lacked an understanding of the communities they serve, and the reality of being a CHW who is trying to meet those needs. CHWs expressed that the lack of understanding of community needs has led to misinterpretation or even blaming of community members, explaining, ‘You never even know what they need or was lacking or why there's a barrier to healthcare, why they're not showing up to their appointments. You don't know that they don't have transportation or they don't have food, or it could be the fact that they can't read or write. You don't know, so they have to build their rapport with clients just like we do in order to be able to provide those services’. CHWs described their perceptions that leaders have education but not real experience with the community. One CHW said, ‘You could get all the education you want and have a PhD and be the top, but you will never understand that because you don't live it, because your family didn't experience it, and because you're not in that person's skin’. Other CHWs described this as a lack of understanding and a disconnect between the community's needs and administrative decisions, stating, ‘I think that if you haven't had boots on the ground, you don't have the lived experience or actually worked with the clients, that you don't understand the needs of it because you're just back there making administration‐type things’. CHWs stated that even when they liked and appreciated their supervisors, they still felt like their supervisors did not understand the reality of the community. One CHW stated, ‘We love our supervisor, but it's just somebody who understands the culture and the people. Yeah, that would be better’. CHWs recommended that supervisors seek ways to better understand the community to help them be better supervisors. One CHW stated, ‘Actually, the client interaction—listening to them, knowing what their needs are, having them at the table when you're making programmatic decisions’. CHWs recommended that their supervisors attend patient visits alongside them when possible to better understand the work being done by the CHW and people and community being served. One CHW stated, ‘Sometimes it takes—it requires you to go to a home visit. You can't sit in the office all day’. CHWs showed appreciation for supervisors and organizational leaders who were proactive in learning about community needs, stating that when they ‘put on a workshop for an action plan… and the CEO was there, and he got a chance to look and see what was needed in the—some of the counties…He saw what the weaknesses were in the lower‐income communities, and he understood everything’.

### 3.5 Expectations versus Reality

CHWs voiced concerns about how the lack of understanding around their work duties creates challenges and unreasonable job expectations. CHWs voiced difficulty managing the expectations of supervisors for administrative work and the realities of helping people in the community. One CHW stated, ‘I can't do admin role as well as case management role as well as home visits, as well as – now what [the client has] got going on, all of that. Another CHW stated, 'It's really easy for somebody to set up at a desk and look out over the city and say, everything looks like it's doing really well today, because they have no clue what we really do’. CHWs tied their supervisors' lack of involvement with their work as contributing to unrealistic expectations, stating, ‘I sometimes wish that they could be more involved in exactly what we do, other than just giving us expectations, numbers and follow through, and then that's it’. Another CHW stated, ‘Learn to sack the food, and then you can say how much time. You have to learn to sack it. Don't come in saying, “Da, da, da, da. I can do any job that my people that I supervise [do]”’.

CHWs described the difficulty with having inflexible metrics of success and stated, ‘Sometimes it takes a whole day to help just one client’. Other CHWs talked about how their work was not confined within typical work hours, stating, ‘The bad part is when you work 10, 12 hours, and then the company looks at you and goes, “Well, you know you only get paid for 8 hours”’. This CHW went on to state they ‘make sure that the people are getting what they need’. Another CHW voiced, ‘Working hours get in the way of my job’. CHWs were complementary of supervisors who try to understand the ever‐evolving nature of CHW work and offer flexible schedules to accommodate CHWs' clientele. CHWs stated, ‘We have a really excellent boss, very supportive. We work a lot because we're outreach, plus interpreters. He's like, “You need to take off. Take off whenever. I don't want you to get burned out”’. CHWs discussed that even when their supervisors were supportive of taking time off, it was difficult to do so because their workload and needs of their clients were often never ending. One CHW stated, ‘“You need to take off.” I'm like, “Yeah, but I cannot”. Yeah. She always wants me to take care of myself first’.

### 3.6 Experiences of Onboarding Into a New Organization

CHWs stated that one of the major barriers to integration was their experience onboarding into a new organization, describing the experience as confusing without a systematic process. One CHW stated, ‘When I first started, I remember I didn't really have a proper training in the beginning. It was more of telling me what to do and telling me how to do it and then just expect me to go out there and do it. That was very hard for me because I had to navigate all of this on my own’. Another CHW stated they were ‘just being thrown out there to navigate everything myself’, and another CHW stated, ‘It wasn't enough training in the beginning. I felt really lost’. CHWs stated the lack of onboarding meant they did not understand how they fit within the larger organization. One CHW stated, ‘It's like we're trying to connect all of these different pieces, and nobody's really meshing well’. CHWs went on to state how the lack of onboarding meant they did not understand the other programs within the organization. One CHW stated, ‘I don't know too much about the other programs or the other things that they are doing’.

### 3.7 Open Communication With Supervisors

CHWs identified open communication with their supervisors as one of the primary facilitators of CHW success. One CHW stated, ‘Our supervisor, they usually give us time to speak and state what we see happening in the community and what should be changed, and we always have the voice to say what we believe need to be changed’. Another CHW expressed, ‘The supervisor I work with, they give me the time to voice what I think will work and will not work out in the community… because I totally believe we're the voice for the community we're working with’. CHWs continuously expressed the importance of supervisors listening to them, stating, ‘It felt like [supervisor] was hearing us out… At least, she was part of the conversations’. Other CHWs mentioned that cultural differences made it difficult to speak openly with their supervisor, stating, ‘In different communities…coming up with a question that does not affect the relationship between leadership and staff, it's hard to do it in some cultures. For example, where I was taught when I was little never to talk back to my parents; always look down speaking to my grandparents. You especially don't tell your boss what's wrong or what's right’.

### 3.8 Comradery, Collaboration, and Co‐Learning

CHWs described comradery and co‐learning within their team as one of the most significant facilitators to integration. One CHW stated, ‘It is like a family because you spend a lot of time with those people. They know your habits. You can't hardly fool them because you with them every day. They know when you're feeling good, when you're feeling bad or when you're trying to fake something’. CHWs tied the comradery within their teams as a primary facilitator of integration and retention, stating, ‘I mean, I like it. I love it. That's why I'm still here, because we all work together as a family. It's like a family to me, and we all get along. We come together, we help each other when somebody's not here. We help each other’.

CHWs reported a feeling of acceptance with team members who mirror their race/ethnicity and expressed appreciation for the diversity in backgrounds and perspectives. CHWs specifically highlighted their enjoyment in working with diverse team members, stating, ‘I think it is the first office that I felt was representative of the community I grew up in’. CHWs connected the comradery to collaboration and a culture of co‐learning. One CHW stated, ‘The fact that our team is willing to be collaborative and have these collaborative conversations just empower more for what we're doing, and I think that really shows for the community. Because if we didn't work well together, again it wouldn't come off authentic, and I don't think people would be trusting it’. CHWs described continuously learning from their team members as a primary facilitator of integration. One CHW stated, ‘I'm learning something from a coworker or from a supervisor because I might not have thought of that. Or I might not have seen it the same way you do’. Another CHW explained, ‘We come from different backgrounds and different points of view. We all bring something to the table’. Other CHWs agreed, stating, ‘I've had a lot of help from my coworkers. They really helped me a lot with navigating everything’.

## 4 Discussion

Arkansas has experienced rapid growth in the CHW workforce [51], and as recent legislation passed requiring Medicaid reimbursement of CHWs, CHW growth in Arkansas is expected to accelerate. This study used a parallel multi‐methods assessment to understand CHWs' perceptions of the factors affecting CHW integration into care teams. In the quantitative survey, CHWs reported a high level of support and integration. The focus groups provided an opportunity for CHWs to discuss factors that increased or hindered their successful integration. While the vast majority of CHWs reported a high level of integration in the quantitative responses, the responses to the qualitative interview centered around barriers and opportunities for improved integration. The lack of congruence in the quantitative and qualitative results provides greater depth into the complexities of CHW integration and demonstrates the value of using both quantitative and qualitative methods.

In the qualitative interviews, CHWs described a lack of role clarity as a significant barrier. CHWs also described the need for better onboarding processes when starting a new position. This finding is consistent with prior literature which documents ambiguity and a lack of understanding surrounding CHWs' roles and responsibilities [46, 47]. It is one of the first studies to provide qualitative data allowing CHWs to describe their needs in their own words. To address this barrier, organizations should consider policies and practices to ensure clear job descriptions and a thoughtful onboarding process that allows CHWs to understand their roles within the organization and the roles of the other organizational units.

CHWs identified organizations' greater involvement with the community as a significant facilitator. CHWs identified challenges when the CHW's organizations lacked an understanding of the community's needs. CHWs are often brought into an organization precisely because of their in‐depth knowledge of the community [59]. Our findings highlight CHWs' recommendations that in addition to relying on CHWs' understanding of community needs, organizational leaders should consider practices that focus on engaging with the community.

One of the primary barriers expressed was the difference between expectations for a CHW and the reality of being a CHW. CHWs reported that their supervisors often had unrealistic expectations. CHWs were most complementary of supervisors who appreciated unpredictability of CHW work and provided flexible schedules. This finding is consistent with prior literature which has documented the lack of understanding of CHW work and afterhours schedules as one of the most challenging parts of being a CHW [29, 45]. This finding may point to a lack of organizational and supervisor readiness. Organizational leaders should consider organizational policies and practices that include CHW‐specific metrics for success and flexibility.

One of the greatest facilitators identified was open communication with supervisors; however, CHWs described how their cultural backgrounds, which often prioritize respecting those in authority, sometimes made communication with their supervisors difficult. While several prior studies have documented that many CHWs are from non‐majority cultures within the US, this study is the first to highlight how CHWs' cultural positionality might affect organizational communication. Organizational leaders should consider policies and practices that encourage open communication and feedback between CHWs and their organizations.

CHWs from organizations who had multiple CHWs described their team as a family and described camaraderie, collaboration, and co‐learning as a primary facilitator of integration. CHWs highlighted this as a significant motivating factor that influenced their connection with the organization and their satisfaction with their work. Organizations that employ CHWs should seek ways to foster camaraderie, collaboration, and co‐learning among CHWs and between CHWs and other professionals.

Organizations can promote integration of CHWs through consistent, structured opportunities for CHWs and other professionals to engage in shared learning, reflection, and problem‐solving. Bidirectional training, interdisciplinary team‐based work, and joint onboarding can further support mutual understanding and respect across roles. These strategies are most effective when paired with inclusive leadership practices and organizational policies that recognize CHWs' lived experience as expertise and unstructured work hours.

Overall, our findings are consistent with prior studies on CHW integration [45, 46, 47, 48]. This article has a more diverse sample than prior studies and makes a significant contribution augmenting the limited literature on CHW integration. Table 4 provides an overview of the implication of our findings to organizational policy and practice.

### 4.1 Limitations and Strengths

This study has several limitations and strengths. All participants were located in Arkansas, which limits generalizability, but the generalizability is strengthened by the racial/ethnic and gender diversity of the sample. Additionally, the study only includes CHW perspectives on integration and does not include their supervisors' perspectives. The study team is currently conducting a study on organizations' perspectives, which will be published in a subsequent paper. Our parallel multi‐methods approach with both quantitative and qualitative data also strengthens the study. The qualitative approach provides detailed insight from CHWs in their own words, which is essential for understanding the complex real‐world dynamics influencing the integration of CHWs. This approach also allows for practical recommendations for how to improve the integration of CHWs.

## Author Contributions

**Pearl A. McElfish:** conceptualization, formal analysis, funding acquisition, investigation, methodology, writing – original draft, writing – review and editing. **Sara Sorrell:** conceptualization, formal analysis, methodology, writing – original draft, writing – review and editing. **Luis Paganelli Marin:** conceptualization, formal analysis, methodology, writing – original draft, writing – review and editing. **Judy Pile:** conceptualization, methodology, writing – review and editing. **Anna Huff:** conceptualization, methodology, writing – review and editing. **Bonnie Faitak:** conceptualization, methodology, writing – review and editing, project administration, supervision. **Sarah Moore:** conceptualization, methodology, writing – review and editing, project administration, supervision. **Amy Ayala:** formal analysis, writing – review and editing. **Sergio Bonilla:** conceptualization, methodology, writing – review and editing, project administration. **Carolina N. Vargas:** conceptualization, methodology, writing – review and editing, project administration. **Manuel E. Tejada:** conceptualization, methodology, writing – review and editing, project administration. **Nicole Thornton:** methodology, writing – review and editing. **Krista Langston:** conceptualization, methodology, writing – review and editing, project administration, supervision.

## Ethics Statement

The study was approved as exempt by the University of Arkansas for Medical Sciences Institutional Review Board (#275076). Participants gave verbal consent for the qualitative interviews and electronic consent for the survey.

## Conflicts of Interest

The authors declare no conflicts of interest.

## Data Availability

The deidentified data underlying the results presented in this study may be made available upon request from the corresponding author, Dr. Pearl A. McElfish, at pamcelfish@uams.edu. The data are not publicly available in accordance with funding requirements and participant privacy.
